# Case of Ovariotimy

**Published:** 1878-09

**Authors:** 


Case of Ovariotomy.—Miss K. B., aged twenty years, was seen by me in consultation with Dr. C. C. Holmes, of Milton, December 11, 1877. At that time her umbilical girth was thirty-three and a half inches, temperature 100.6° Fh., pulse 132, of fair strength, and her general condition encouraging for operation. Dyspnœa and general discomfort had induced Dr. Holmes to tap the cyst on December 5th, when twenty pints of clear, yellow fluid were removed.

Her health first began to fail in March, nine months before I saw her. On March 19, 1878, twenty-eight pounds of brownish-colored fluid were removed by aspiration, and soon after this the patient decided to have the tumor removed. This was done at the Carney Hospital on March 30th, under carbolic acid spray. The incision was five inches long, and subsequently was somewhat enlarged; there was a slight amount of ascitic fluid; the adhesions were strong, quite vascular, and almost universal throughout the anterior and lateral portions of the cyst wall, and to the diaphragm on the left side they were particularly intimate and difficult to separate. Many of the bands of adhesion were tied in two places with silk or cat-gut, and divided between the knots; others were cut through with saw-toothed scissors, and many were ruptured by manual force. A small laceration of the surface of the uterus, which bled freely, was sewed up with carbolized cat-gut. The pedicle was first compressed with Dawson's clamp, then tied in two halves with carbolized cat-gut, cut off, and dropped back. This method I have adopted in my last two successful cases, and am very well satisfied with the results. The cyst was extremely multilocular, and the inner cysts were broken down with considerable difficulty. The contents of one of them was shreddy-looking, (very likely this was the large cyst aspirated about two weeks before). The cavity of the abdomen was thoroughly sponged out, and the inner surface of the peritonæum was left red and shaggy-looking. Seven deep and three superficial carbolized silk sutures closed the wound. The patient rallied well from the operation. The pulse rose to 140 on the morning after the operation, and fell to 100 on the third day; the temperature rose gradually to 103.2° on the second day, and was normal on the fifth; the urine was passed voluntarily throughout the period of recovery; at first micturition was frequent (once an hour); flatus passed per anum on the third day; small doses of opiates and stimulants were required; the stitches were well removed on the seventh day, and the wound found to be healed throughout without suppuration. The bowels moved naturally on the eighth day; patient sat up on the fifteenth day, was out of doors on the eighteenth, and returned home, driving seven miles in a carriage, on the twenty-eighth day. The catamenia appeared in May, and have recurred regularly since. She is now (July 22, 1878) well and strong.—*Boston Medical and Surgical Journal.*

